# Supplementary figures and images for: Study of Insulin Aggregation and Fibril Structure under Different Environmental Conditions
Source: Int J Mol Sci. 2024 Aug 29;25(17):9406. doi: 10.3390/ijms25179406 (PMC11395423; doi:10.3390/ijms25179406)

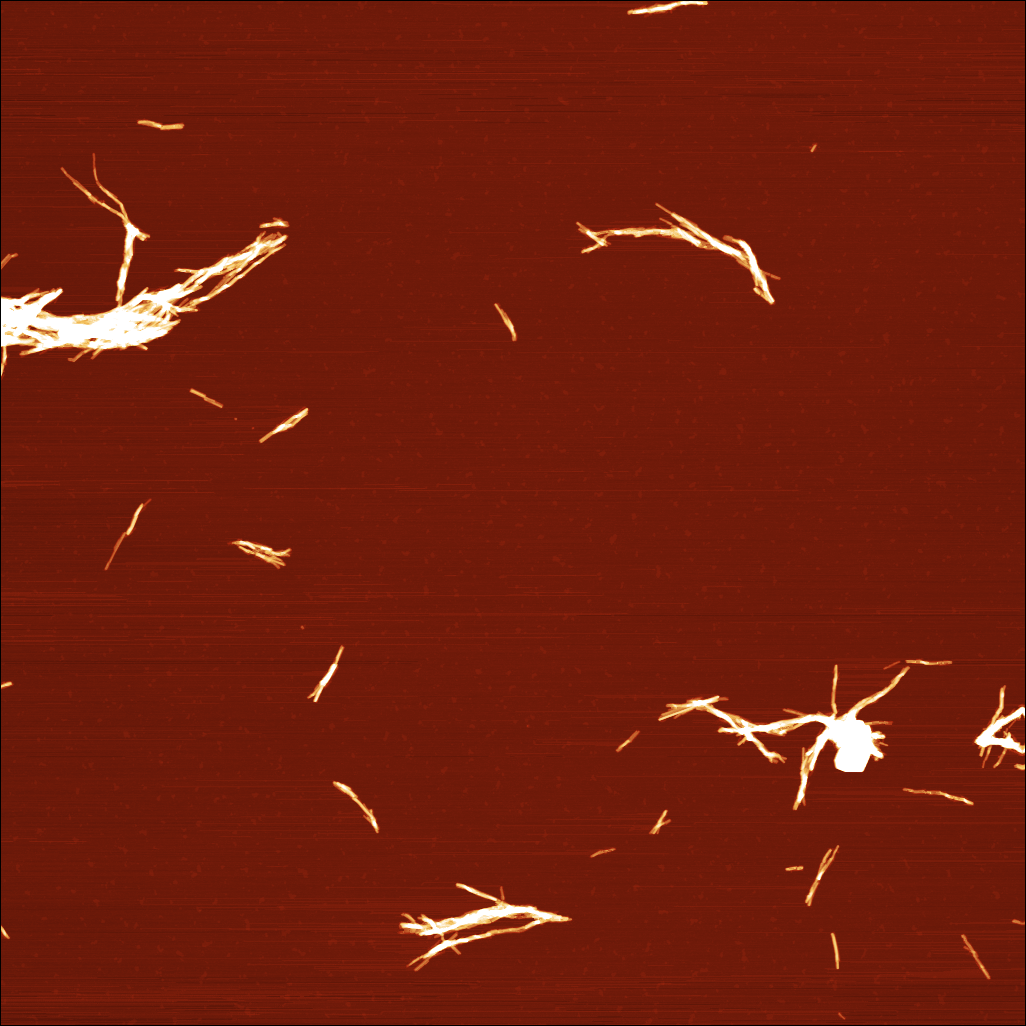

Supplement: Supplementary file 1 [file ijms-25-09406-s001.zip › Ac 100mM.tiff]

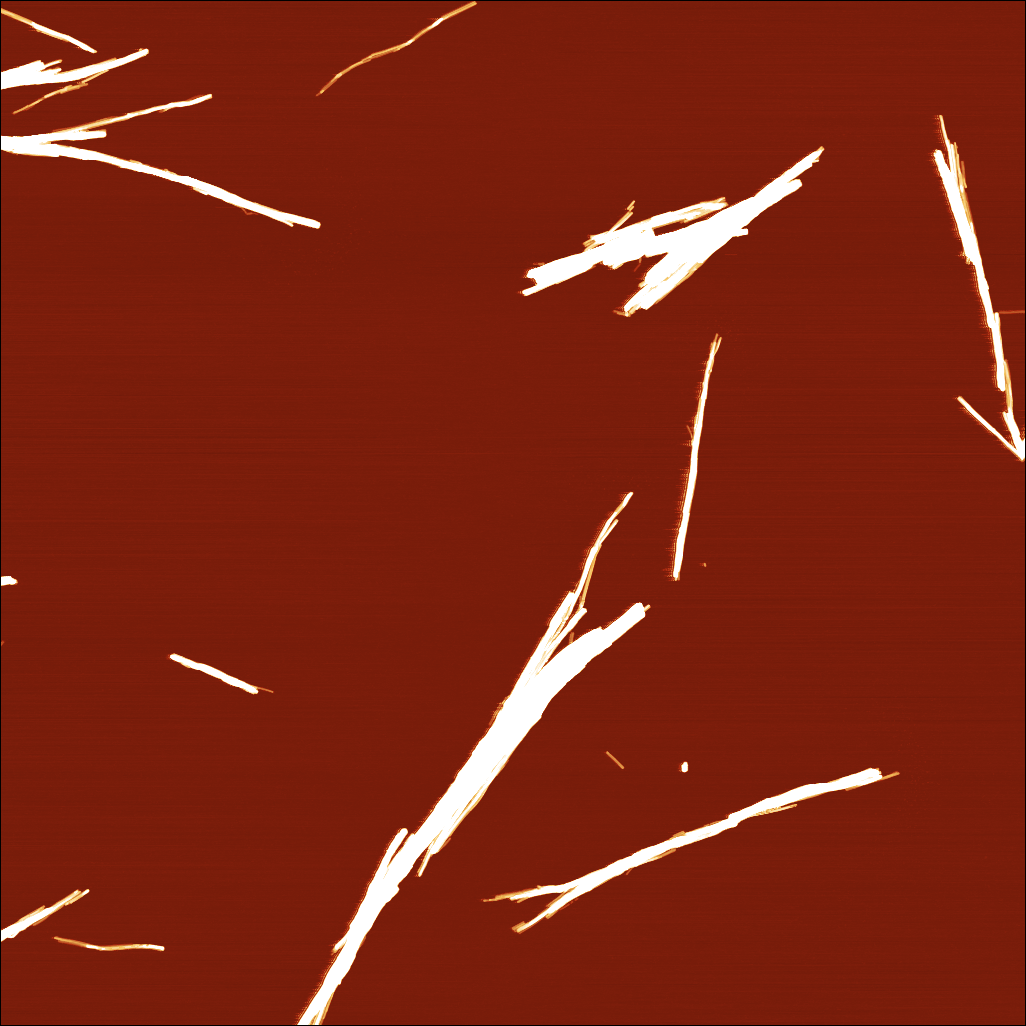

Supplement: Supplementary file 1 [file ijms-25-09406-s001.zip › Ac 300mM.tiff]

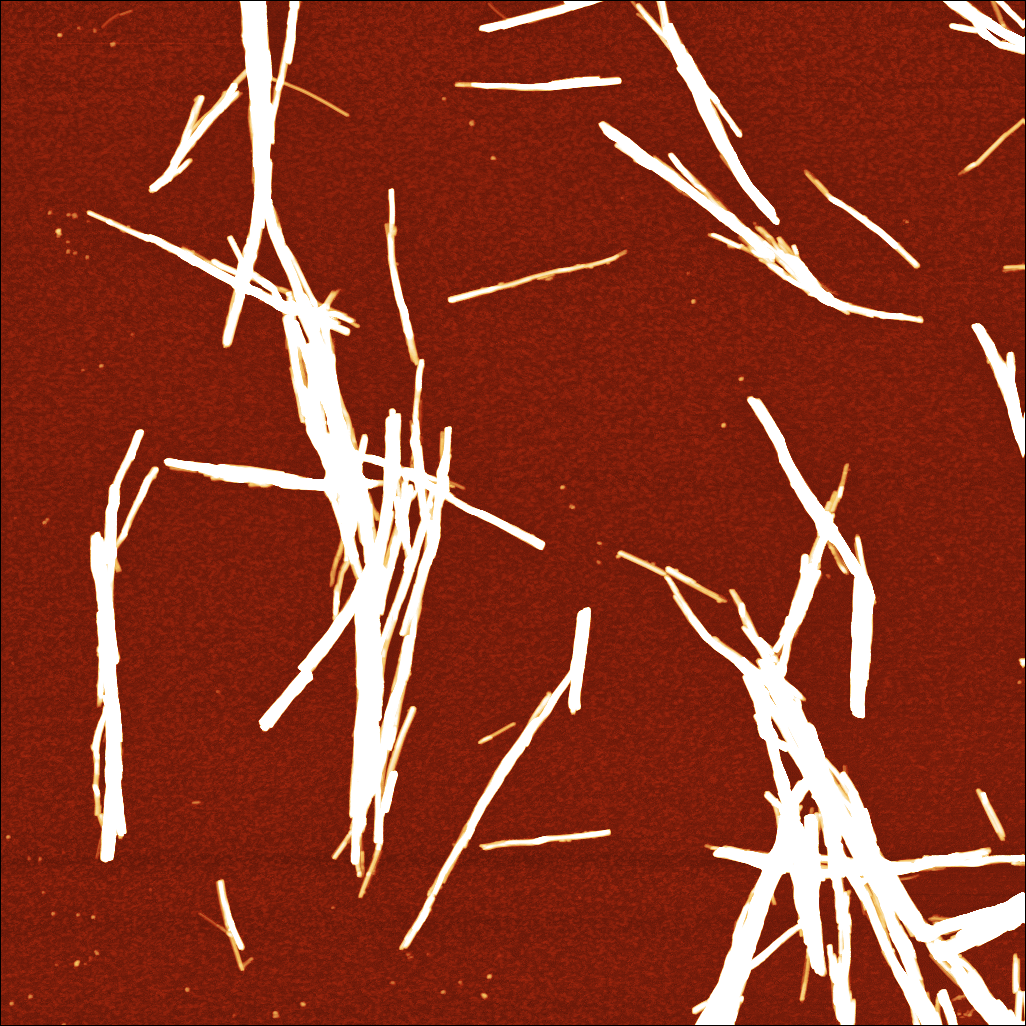

Supplement: Supplementary file 1 [file ijms-25-09406-s001.zip › Ac 500mM.tiff]

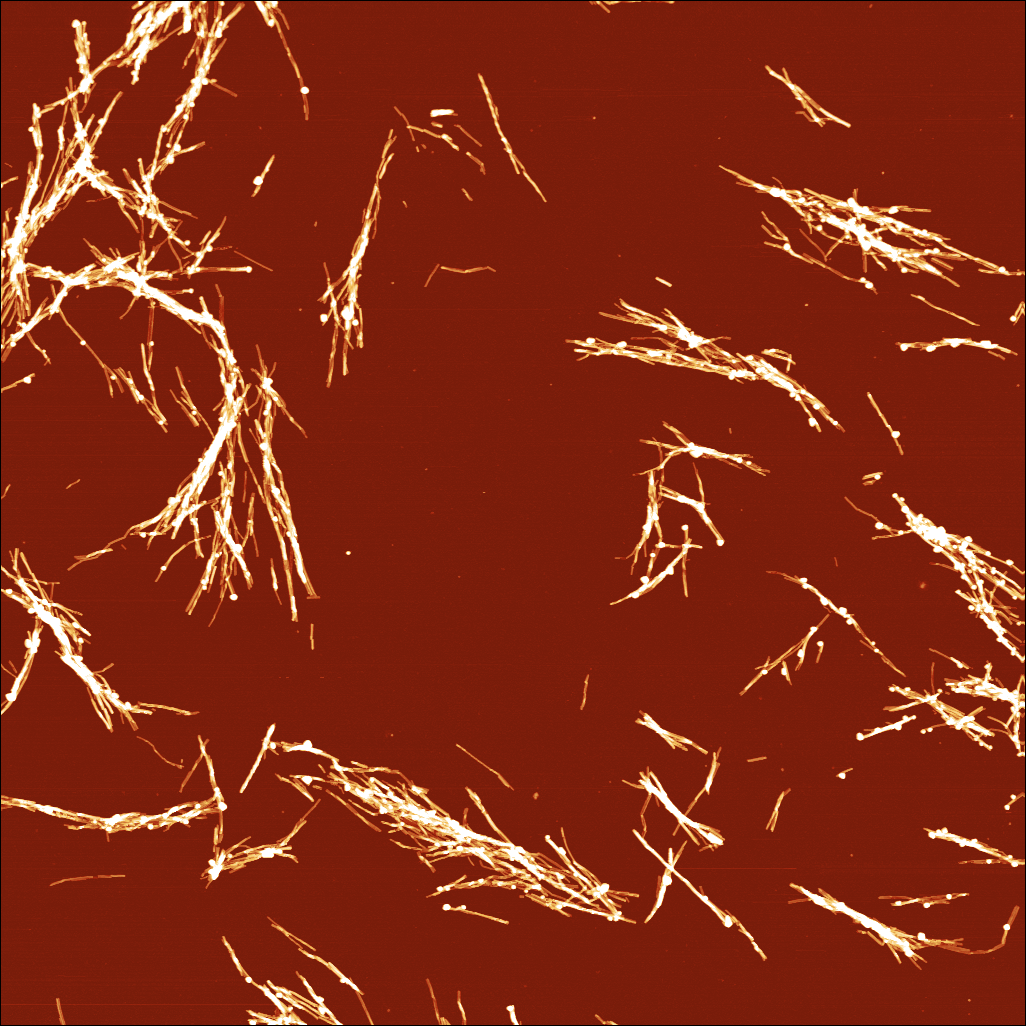

Supplement: Supplementary file 1 [file ijms-25-09406-s001.zip › HCl 100mM.tiff]

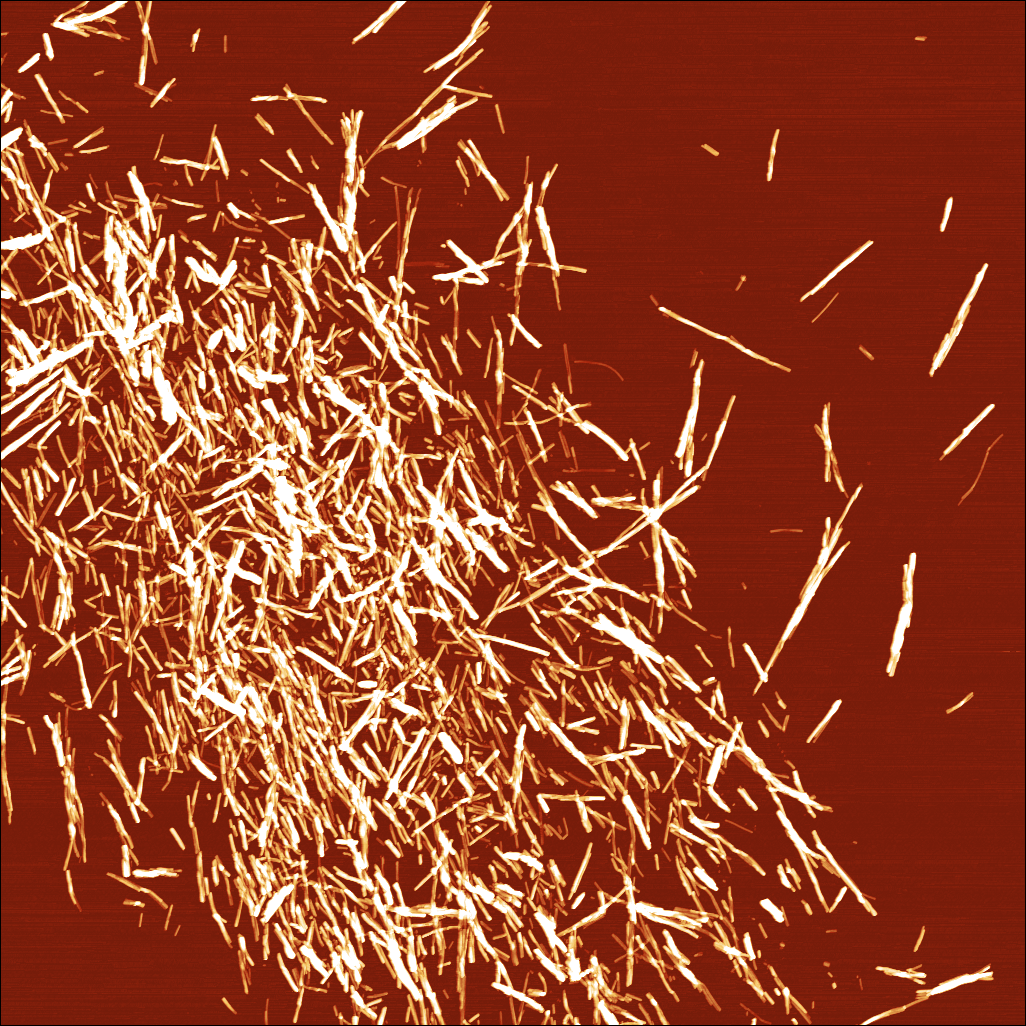

Supplement: Supplementary file 1 [file ijms-25-09406-s001.zip › HCl 300mM.tiff]

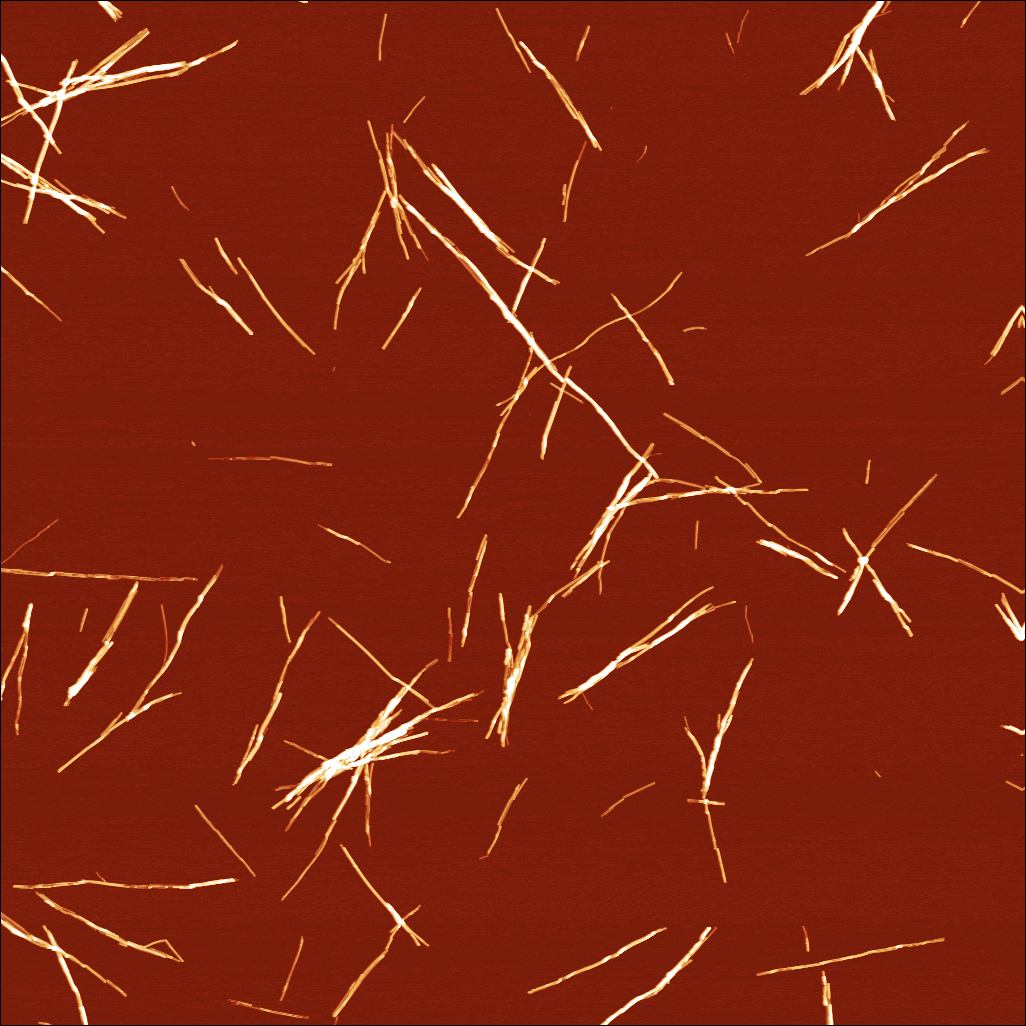

Supplement: Supplementary file 1 [file ijms-25-09406-s001.zip › HCl 500mM.tiff]

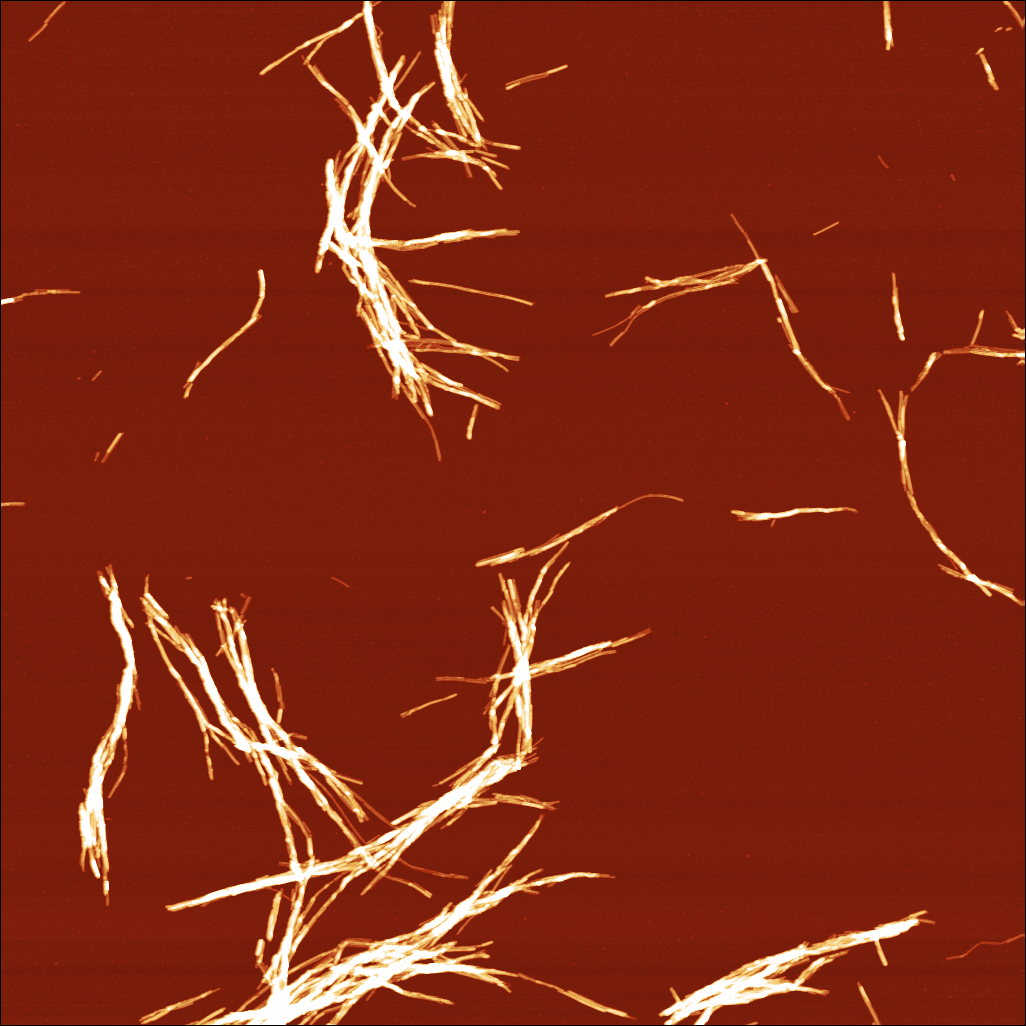

Supplement: Supplementary file 1 [file ijms-25-09406-s001.zip › pH1 100mM.tiff]

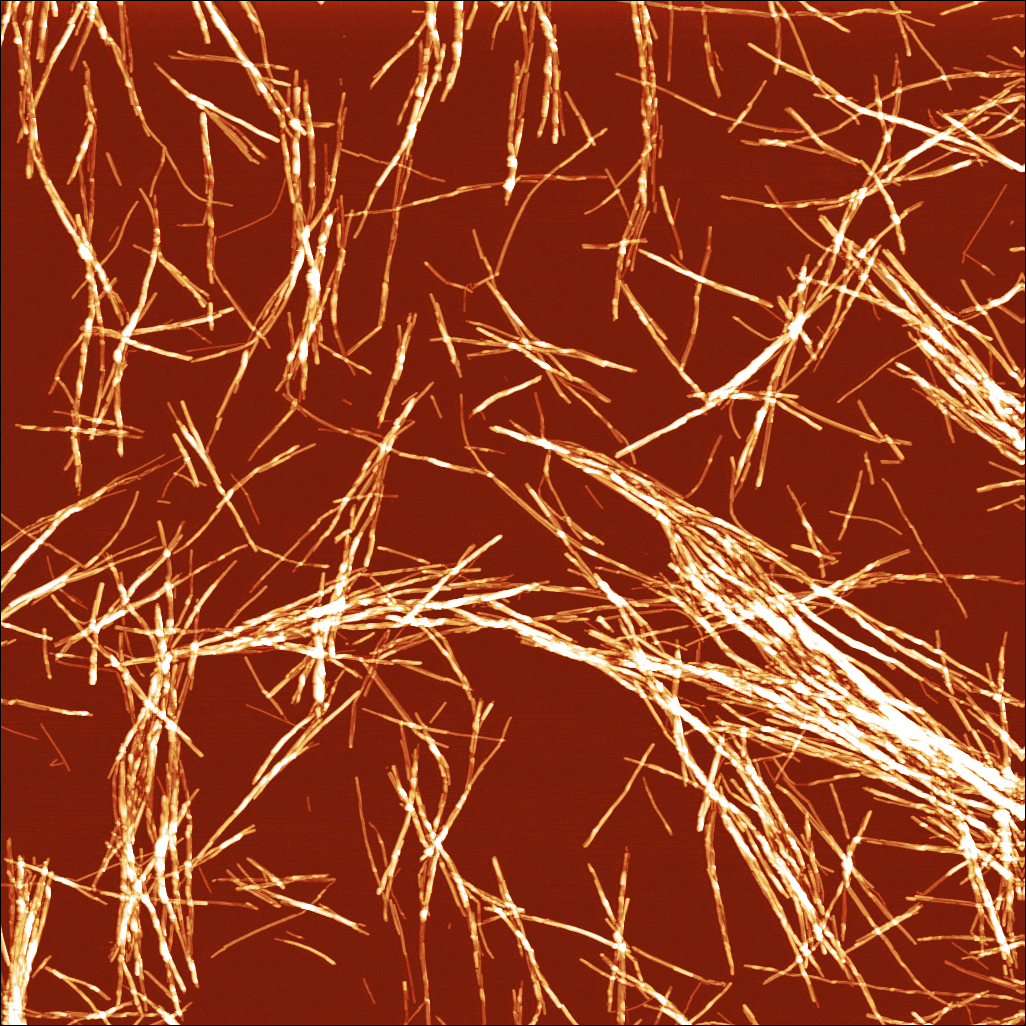

Supplement: Supplementary file 1 [file ijms-25-09406-s001.zip › pH1 300mM.tiff]

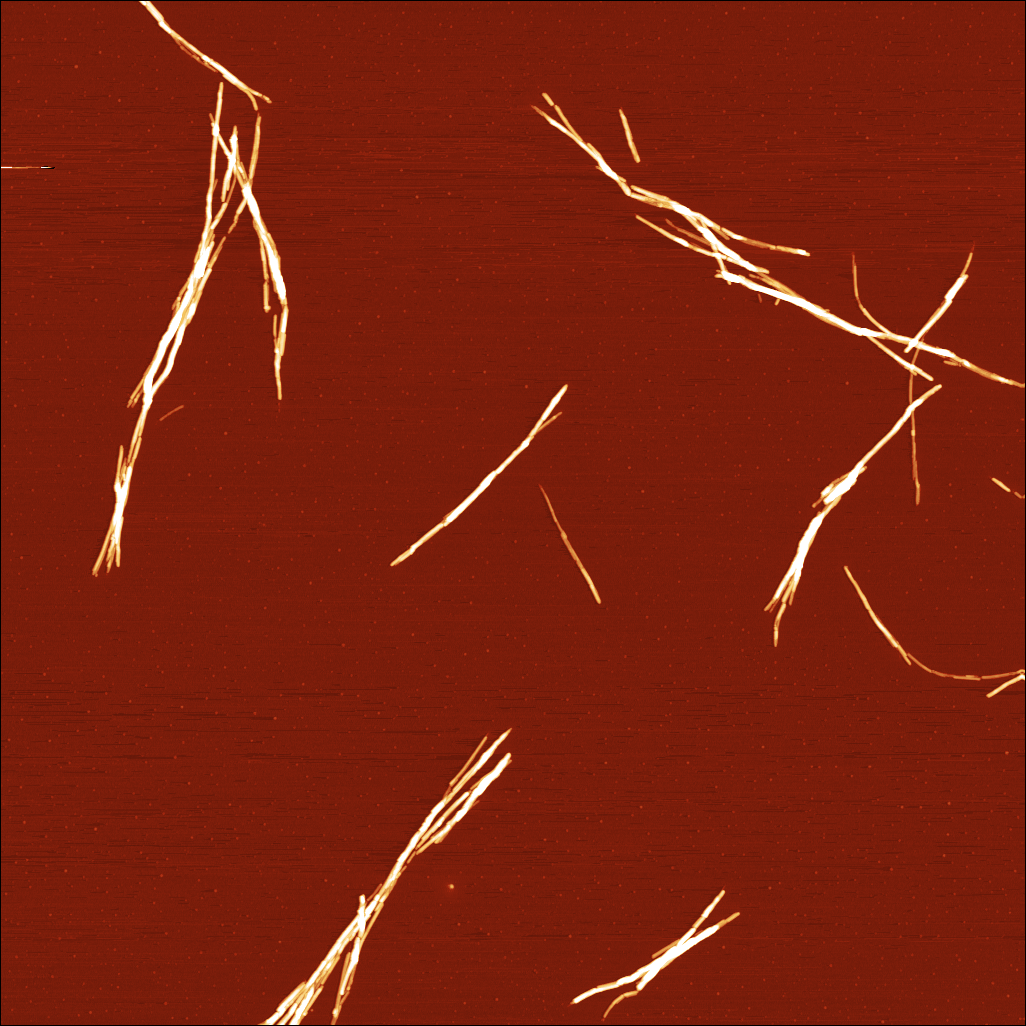

Supplement: Supplementary file 1 [file ijms-25-09406-s001.zip › pH1 500mM.tiff]

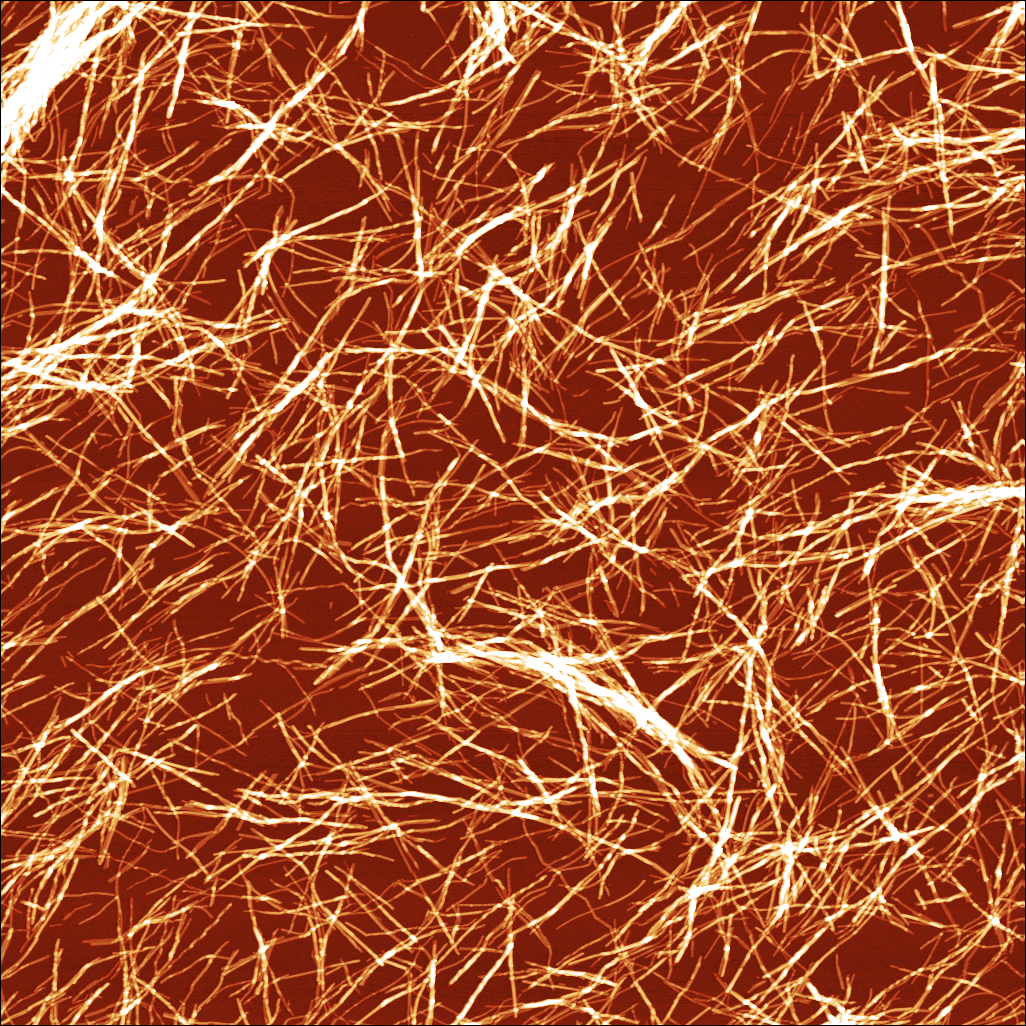

Supplement: Supplementary file 1 [file ijms-25-09406-s001.zip › pH1.5 100mM.tiff]

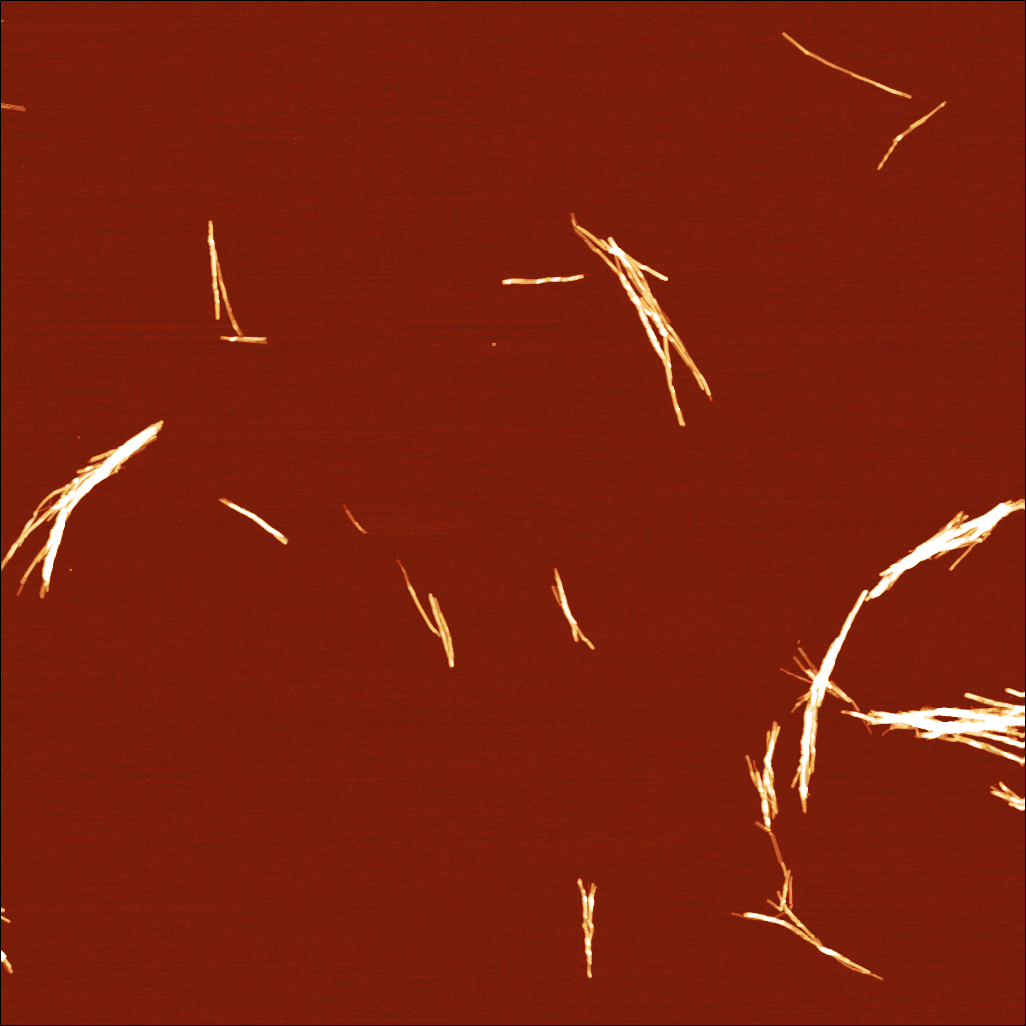

Supplement: Supplementary file 1 [file ijms-25-09406-s001.zip › pH1.5 300mM.tiff]

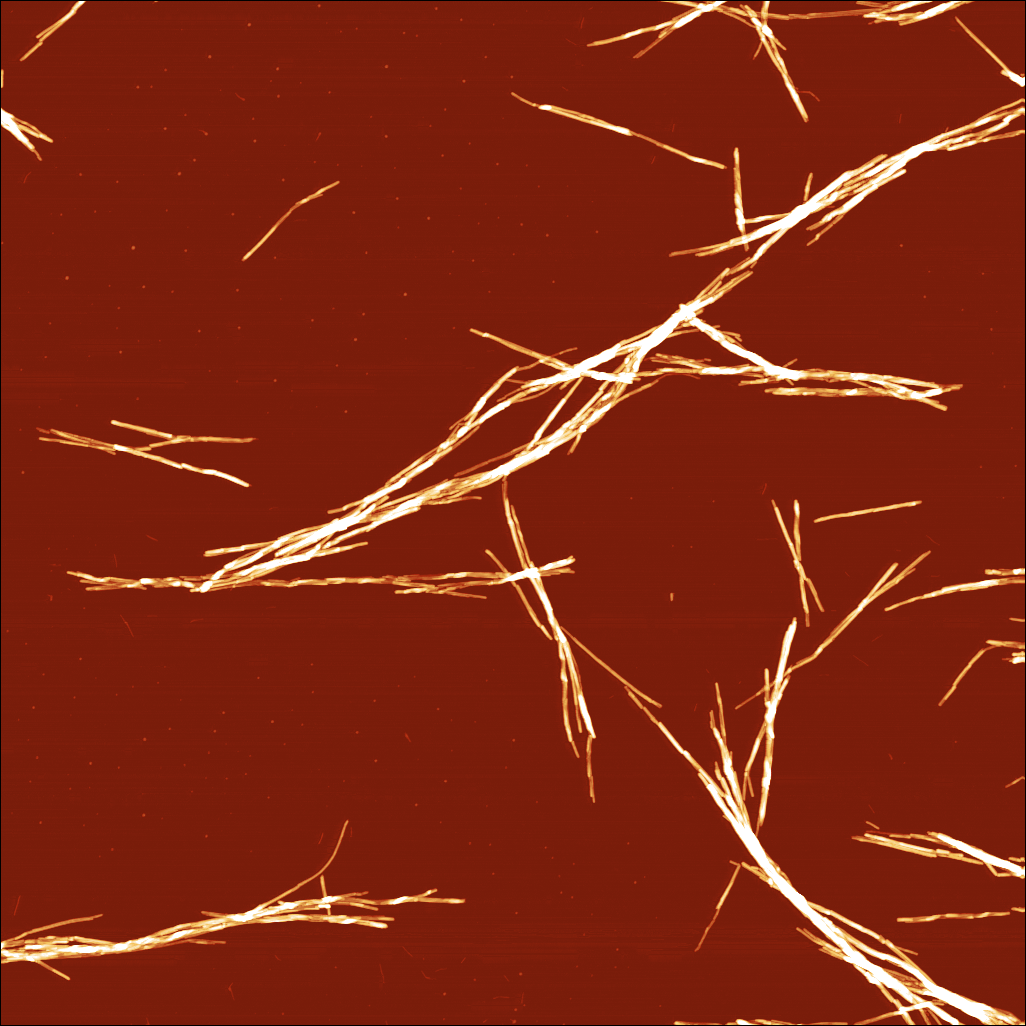

Supplement: Supplementary file 1 [file ijms-25-09406-s001.zip › pH1.5 500mM.tiff]

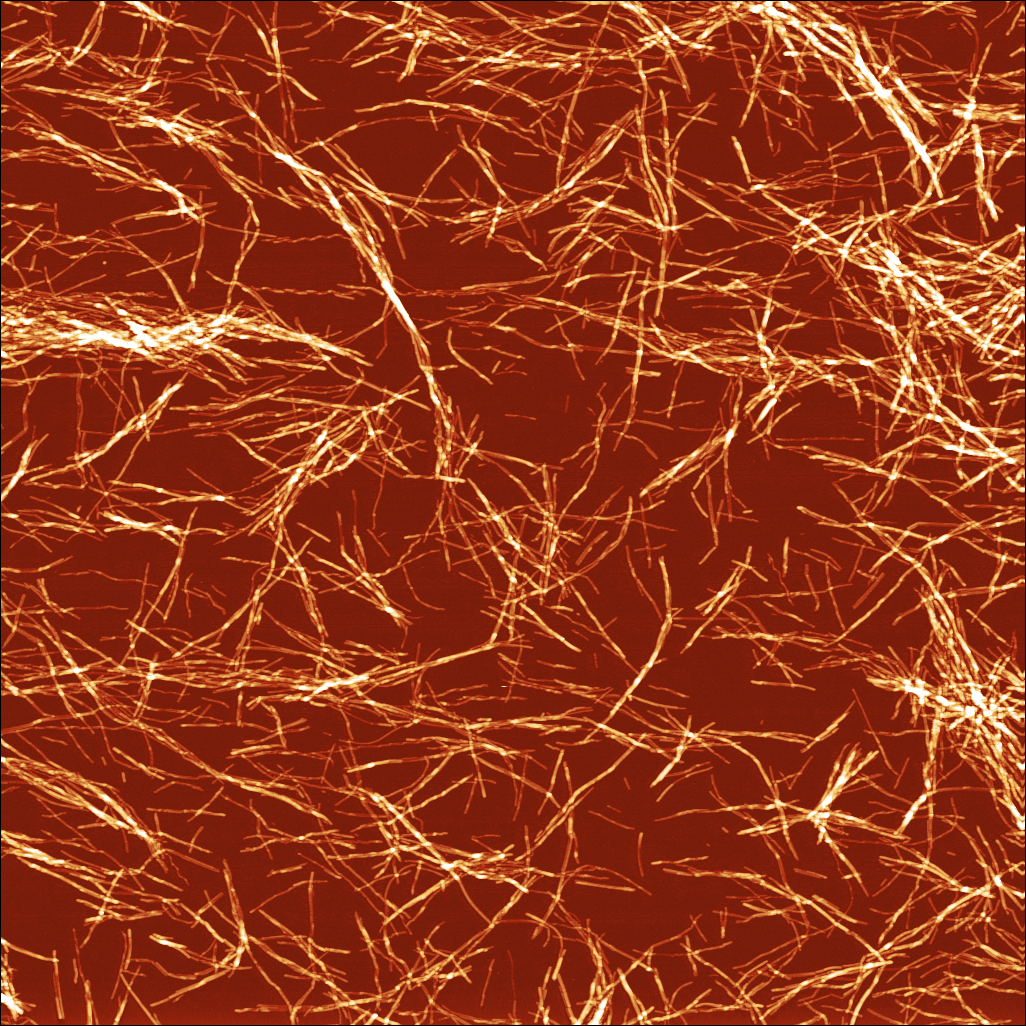

Supplement: Supplementary file 1 [file ijms-25-09406-s001.zip › pH2 100mM.tiff]

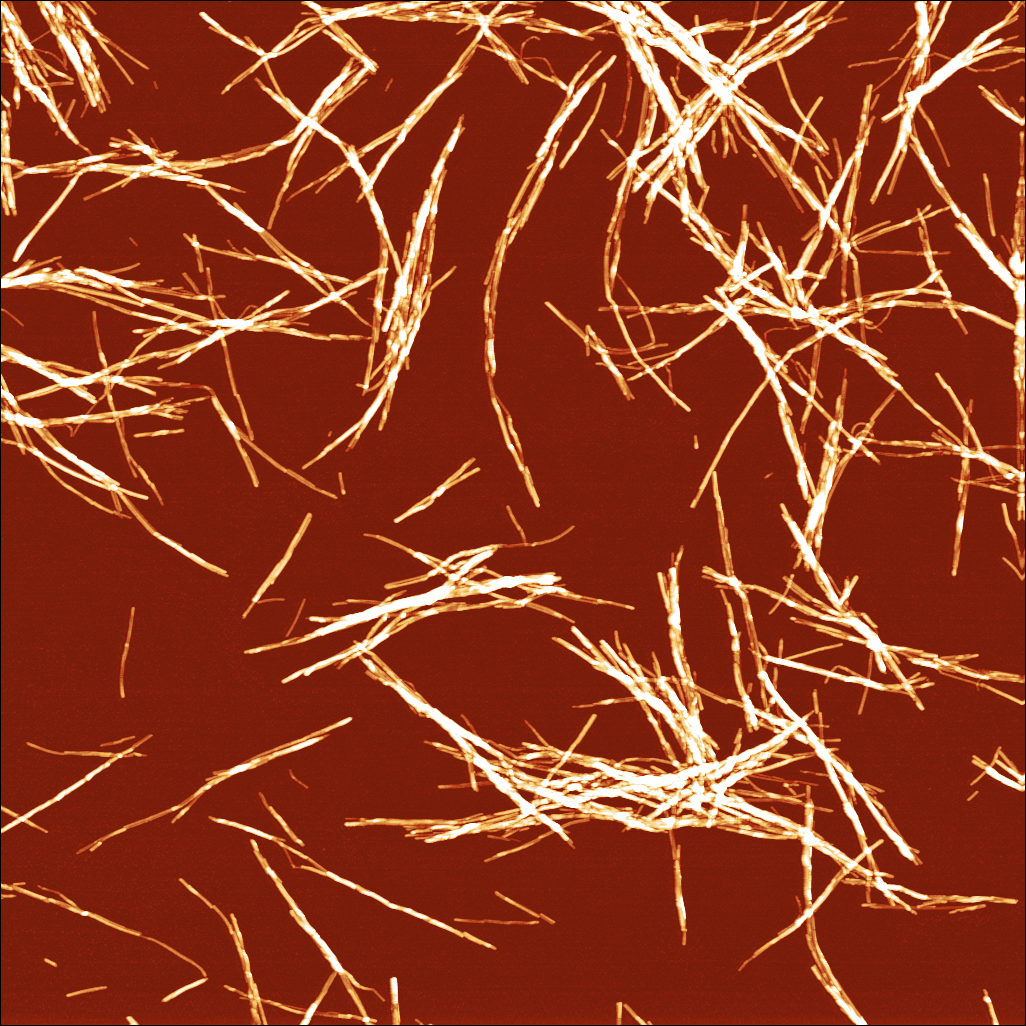

Supplement: Supplementary file 1 [file ijms-25-09406-s001.zip › pH2 300mM.tiff]

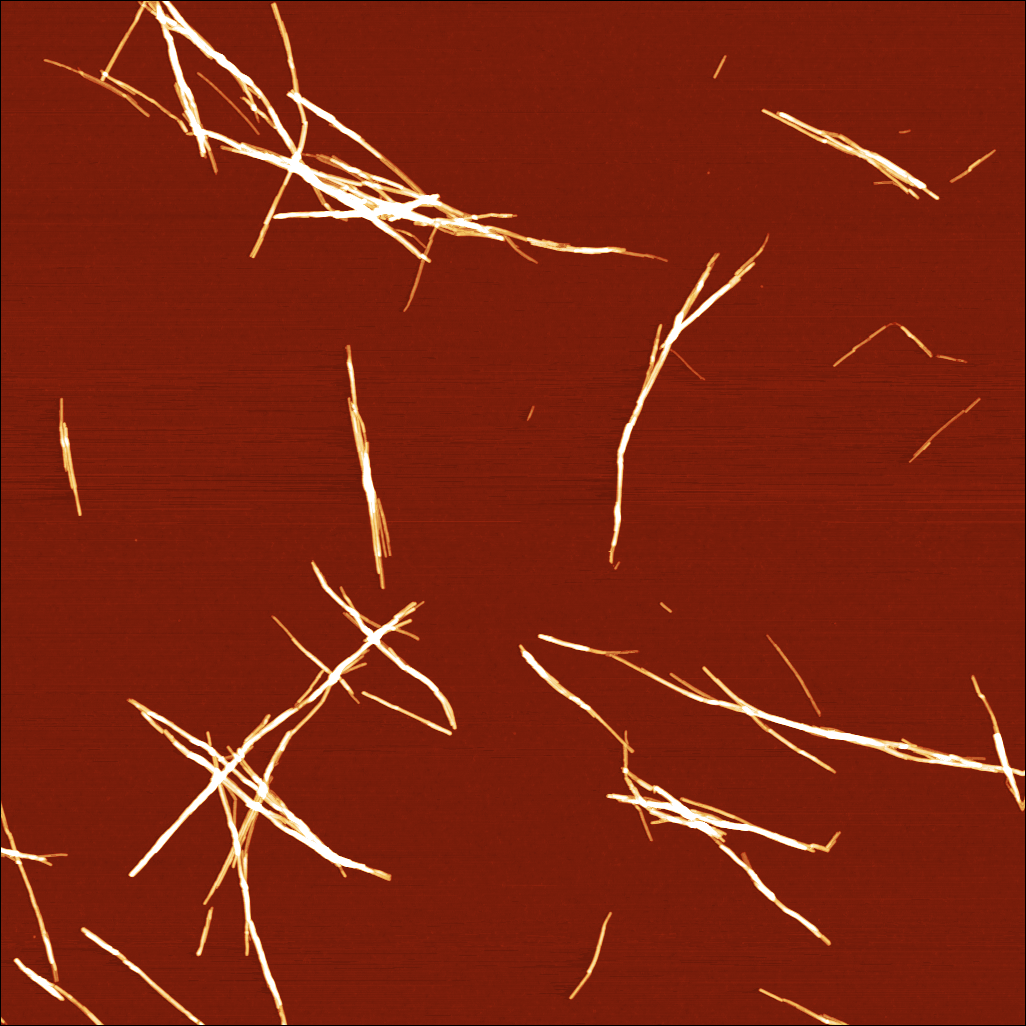

Supplement: Supplementary file 1 [file ijms-25-09406-s001.zip › pH2 500mM.tiff]

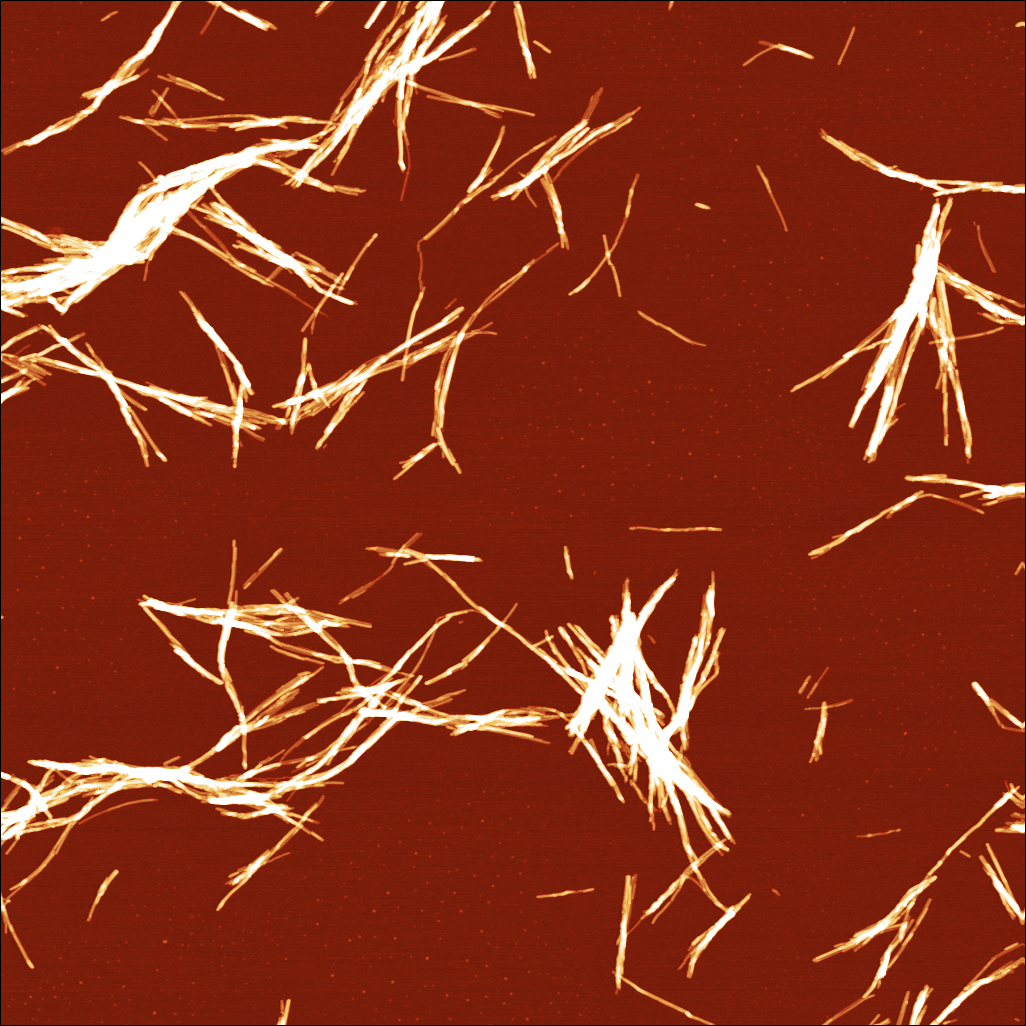

Supplement: Supplementary file 1 [file ijms-25-09406-s001.zip › pH2.5 100mM.tiff]

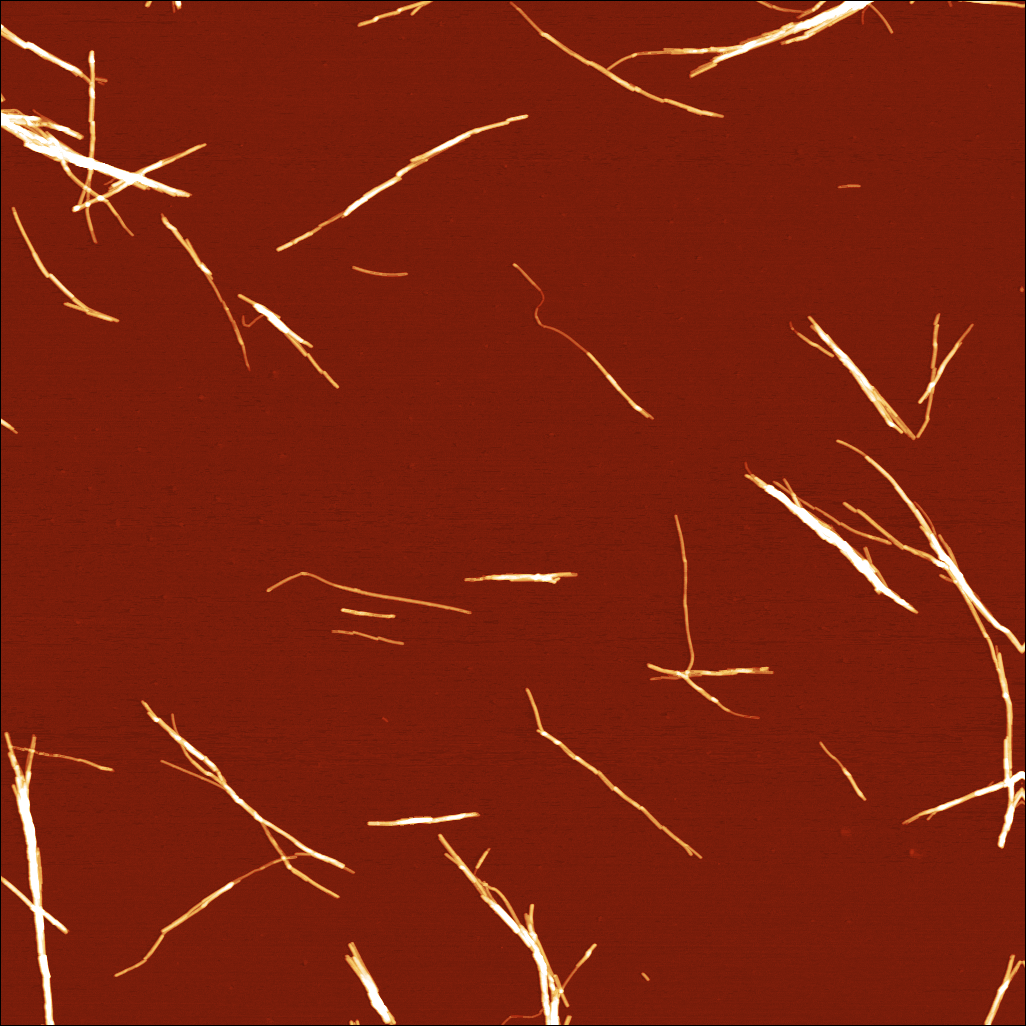

Supplement: Supplementary file 1 [file ijms-25-09406-s001.zip › pH2.5 300mM.tiff]

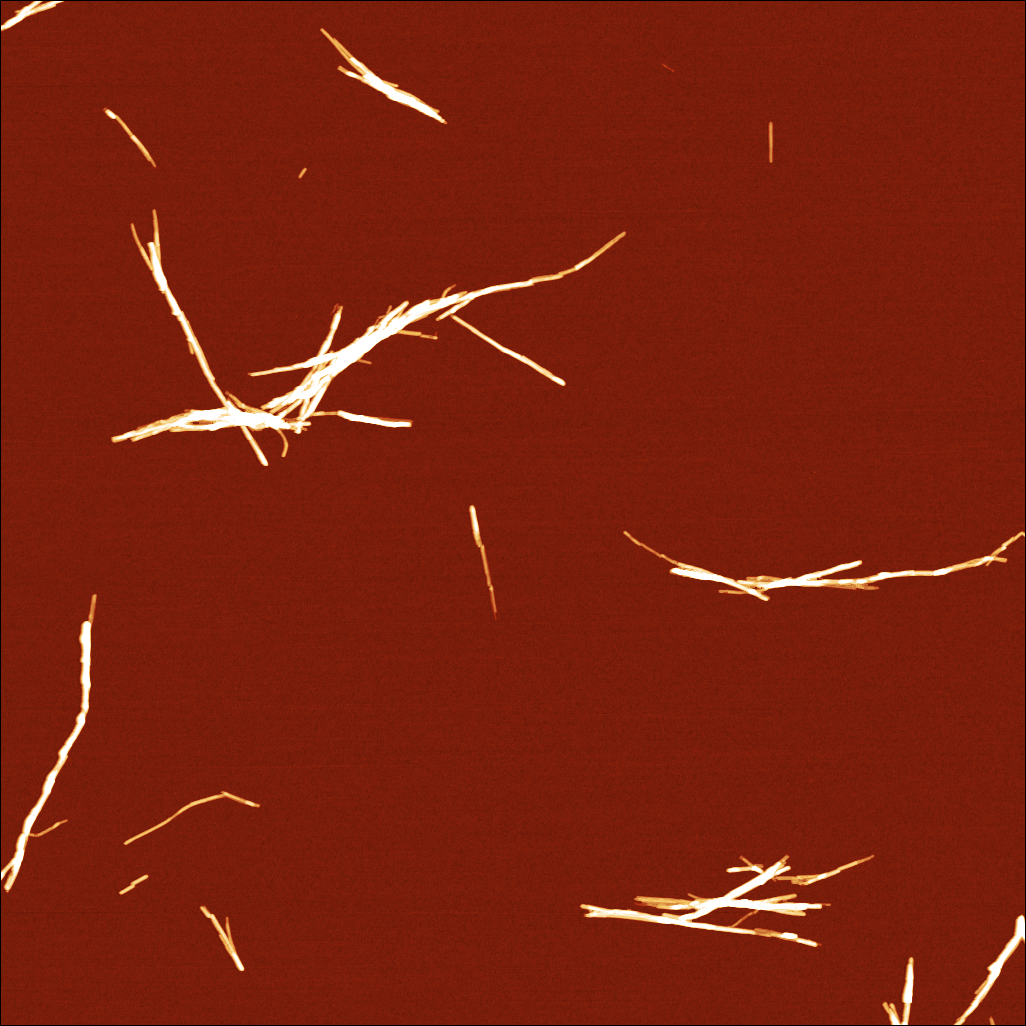

Supplement: Supplementary file 1 [file ijms-25-09406-s001.zip › pH2.5 500mM.tiff]

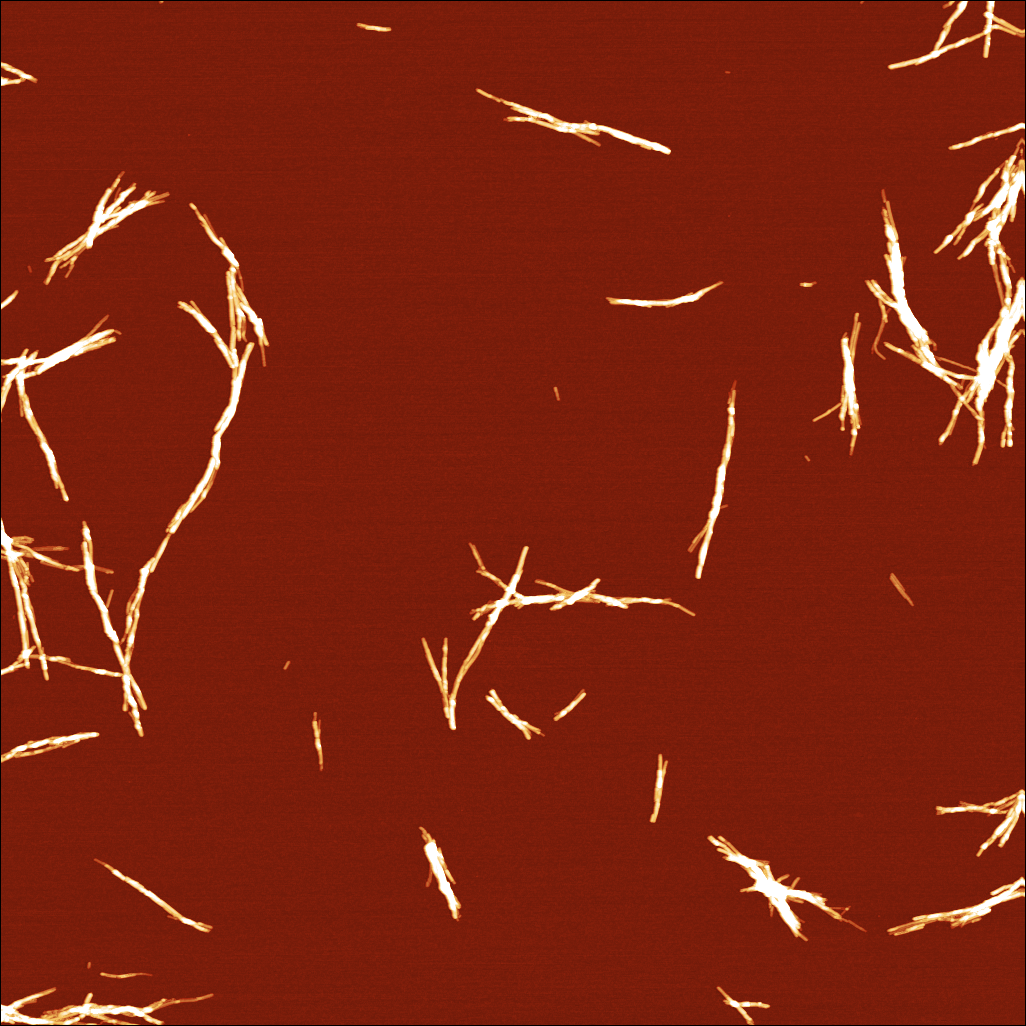

Supplement: Supplementary file 1 [file ijms-25-09406-s001.zip › pH3 100mM.tiff]

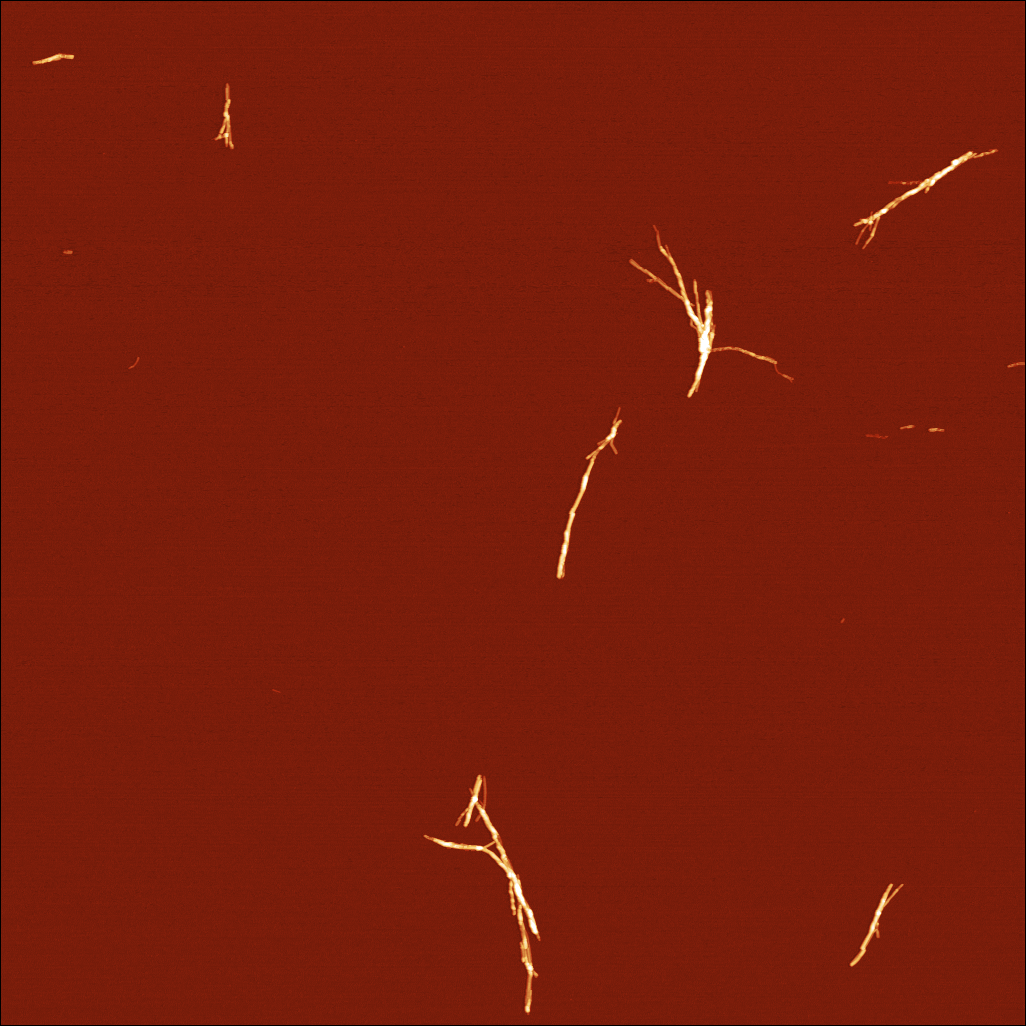

Supplement: Supplementary file 1 [file ijms-25-09406-s001.zip › pH3 300mM.tiff]

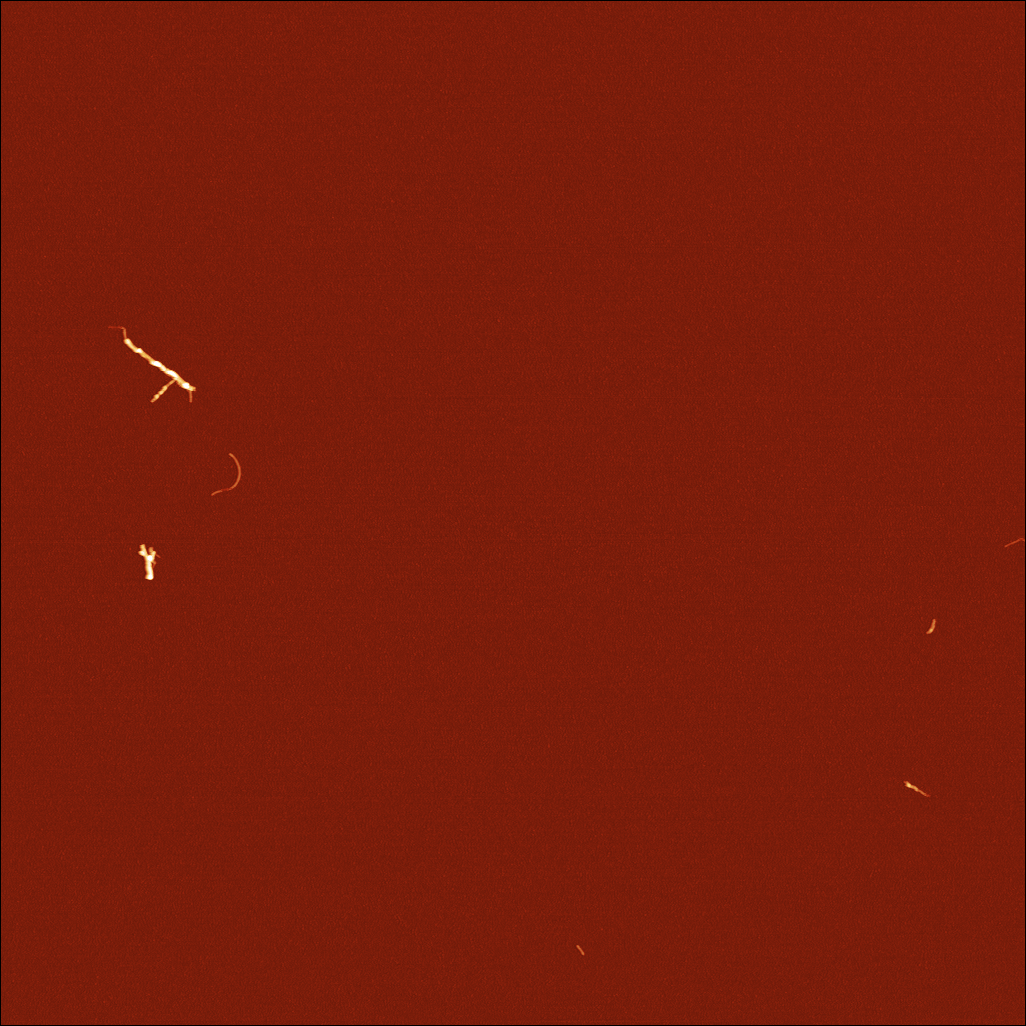

Supplement: Supplementary file 1 [file ijms-25-09406-s001.zip › pH3 500mM.tiff]
